# StackDPP: a stacking ensemble based DNA-binding protein prediction model

**DOI:** 10.1186/s12859-024-05714-9

**Published:** 2024-03-14

**Authors:** Sheikh Hasib Ahmed, Dibyendu Brinto Bose, Rafi Khandoker, M Saifur Rahman

**Affiliations:** Department of CSE, BUET, ECE Building, West Palashi, Dhaka, 1000 Bangladesh

**Keywords:** DNA-binding protein, Sequence identity, Classification, Data imbalance, Recursive feature elimination

## Abstract

**Background:**

DNA-binding proteins (DNA-BPs) are the proteins that bind and interact with DNA. DNA-BPs regulate and affect numerous biological processes, such as, transcription and DNA replication, repair, and organization of the chromosomal DNA. Very few proteins, however, are DNA-binding in nature. Therefore, it is necessary to develop an efficient predictor for identifying DNA-BPs.

**Result:**

In this work, we have proposed new benchmark datasets for the DNA-binding protein prediction problem. We discovered several quality concerns with the widely used benchmark datasets, PDB1075 (for training) and PDB186 (for independent testing), which necessitated the preparation of new benchmark datasets. Our proposed datasets UNIPROT1424 and UNIPROT356 can be used for model training and independent testing respectively. We have retrained selected state-of-the-art DNA-BP predictors in the new dataset and reported their performance results. We also trained a novel predictor using the new benchmark dataset. We extracted features from various feature categories, then used a Random Forest classifier and Recursive Feature Elimination with Cross-validation (RFECV) to select the optimal set of 452 features. We then proposed a stacking ensemble architecture as our final prediction model. Named *Stacking Ensemble Model for DNA-binding Protein Prediction*, or *StackDPP* in short, our model achieved 0.92, 0.92 and 0.93 accuracy in 10-fold cross-validation, jackknife and independent testing respectively.

**Conclusion:**

StackDPP has performed very well in cross-validation testing and has outperformed all the state-of-the-art prediction models in independent testing. Its performance scores in cross-validation testing generalized very well in the independent test set. The source code of the model is publicly available at https://github.com/HasibAhmed1624/StackDPP. Therefore, we expect this generalized model can be adopted by researchers and practitioners to identify novel DNA-binding proteins.

## Introduction

DNA-binding proteins (DNA-BPs) contain one or more DNA-binding domains which enable them to bind and interact with DNA. DNA-BPs are essential for numerous biological processes, such as transcriptional control, genomic rearrangements, replication, repair, modification and so on [1]. These proteins are indispensable for the assortment and separation of single-stranded DNA as well as for the detection of DNA damage. Consequently, proteins that target certain DNA sequences have the potential to be treatments for malignancies and genetic disorders. Transcription factors, nucleases, histones etc. are some other examples of DNA-binding proteins. Transcription factors regulate the transcription process, nucleases cut DNA molecules, and histones are involved in the packaging of chromosomes in the cell nucleus. DNA-BPs exhibit significant sequence and structural diversity. These proteins can be categorized into several families according to their structural motifs, including the helix-trun-helix, zinc finger, leucine zipper, C2-H2 etc. [1, 2]. Unfortunately, the most contemporary approaches to identify DNA-binding proteins possess several shortcomings as a result of overly uneven data. Thus, a rapid and efficient method for identifying DNA-binding proteins is needed. Although numerous works have been published in this area in the last decade, further research is warranted to improve the prediction quality.

Early computational predictors of DNA-BP relied on structural information of the proteins [3–10]. This limits the application of these predictors to proteins with experimentally determined structure. However, for the vast majority of sequence-known proteins, structure is yet to be determined. Therefore, in the past dacade, many new predictors have been proposed that does not rely on true structure of the protein. Wei et al. [11] trained a random forest classifier using local Pse-PSSM (Pseudo Position-Specific Scoring Matrix) characteristics and produced encouraging results. The suggested characteristics could effectively capture local conservation information from the evolutionary profiles along with sequence-order information. Named *Local-DPP*, the model was trained using the PDB1075 benchmark dataset, developed by Liu et al. [12]. On the other hand, PDB186 [13] benchmark dataset was used for independent testing. At the time of its publication, Local-DPP outperformed all the contemporary methods both in jackknife and independent testing. Notably, to avoid homology bias during independent testing, they removed sequences from PDB1075 which had more than 25% sequence similarity with sequences in PDB186. They then retrained the model in the reduced training set to perform the independent testing. Most of the subsequent models using the same benchmark datasets have followed their approach.

Chowdhury et al. introduced iDNAProt-ES [14], a DNA-binding protein prediction approach that makes use of both sequence-based evolutionary information and predicted structure-based properties of proteins. In their study, they leaned on properties like bigram, Position Specific Scoring Matrix (PSSM) composition, and secondary structure occurrence to reach their desired outcome. They extracted such features using PSI-BLAST [15] and SPIDER2 [16], then applied recursive feature elimination, followed by model training using SVM with a linear kernel. iDNAProt-ES also used PDB1075 for training and PDB186 for independent testing. While it outperformed Local-DPP in jackknife as well as independent testing, it was later discovered that their independent testing had a flaw resulting in more than 25% sequence similarity between the training and the test sets, thereby invalidating the independent test results [17].

Another technique, known as the *DNA-binding Protein Prediction model using Chou’s general PseAAC (DPP-PseAAC)* [17], derived relevant information from protein sequences without relying on functional domain, structural or evolutionary information. The authors used Random Forest (RF) to rank the features after feature extraction. They then utilized the Recursive Feature Elimination (RFE) approach to extract an optimum set of features before training a prediction model with a linear kernel using Support Vector Machine (SVM). As DPP-PseAAC does not depend on PSSM or predicted structural features, the model is very fast to train and it can quickly infer prediciton results on novel proteins. It has the best jackknife performance till now in the PDB1075 dataset. However, the independent test performance in PDB186 dataset, while commendable, falls significantly compared to the jackknife results. This suggests overfitting the training set and lack of generalizability to novel datasets.

Nanni et al. [18] developed a representation of proteins based on their 3-dimensional tertiary structure. Their experiment produced a more accurate independent test result for identifying DNA-binding proteins. Fu et al. [19] introduced K-PSSM-Composition, a unique feature creation approach based on PSSM. They too leveraged recursive feature elimination to obtain the ideal collection of features and trained a support vector machine model. Adilina et al. [20] recently developed another approach that retrieved numerous properties such as monogram percentile separation, bigram percentile separation, closest neighbor bigram, etc. solely using the protein primary sequence. They applied grouped feature selection as well as recursive feature elimination for selecting features. Extra Tree and Random Forest classifiers were trained to produce the final prediction models. Hu et al. [21] applied deep learning to solve the DNA-BP classification problem. They attempted to identify the functional domain of the protein sequence by combining CNN with a Bidirectional LSTM. They prepared a large training dataset to train the deep learning model. However, our investigation raises some concerns about this dataset, which suggests their results may be overestimated (Section “A need for new benchmark datasets”).

From the brief literature review presented above, it is clear that quite a bit of work has been published in recent times to tackle the problem of DNA-BPs prediction. The majority of these predictors have been trained using the PDB1075 dataset and tested on the PDB186. However, our analysis (Section “A need for new benchmark datasets”) has uncovered several concerns about the quality of the PDB1075 dataset. Therefore, in this paper, we have attempted to rectify the issues by preparing a new benchmark dataset. We have also retrained selected state-of-the-art predictors in the new dataset.

The specific contribution of this work can be enumerated as follows.

- We have identified quality issues with the PDB1075 benchmark training dataset that has widely been used to train DNA-BP predictors in recent times. Also, there are several common sequences between this and the PDB186 dataset used for independent testing. We have noted that this has unnecessarily complicated the training and testing process.
- To mitigate this problem, we have prepared new benchmark datasets: UNIPROT1424 (training set), comprising 712 DNA-BPs and 712 non-DNA-BPs, and UNIPROT356 (independent test set) consisting of 178 DNA-BPs and 178 non-DBA-BPs. We have ensured 25% sequence identity threshold within as well as between the sets of DNA-BP and non-DNA-BPs in each of the benchmark datasets. We have also ensured the same threshold within and between the training and test sets.
- We have retrained selected state-of-the-art DNA-BP predictors using the UNIPROT1424 dataset and reported the cross-validation performances. We have also reported independent test performances in the UNIPROT356 test set. These results will help researchers in future in comparing their proposed new DNA-BP prediction models with the state-of-the-art methods.
- We have trained a new stacking ensemble based DNA-BP predictor using the UNIPROT1424 training set and benchmarked it in the UNIPROT356 test set. Named *Stacking Ensemble Model for DNA-binding Protein Prediction*, or *StackDPP* in short, our model achieved 91.86%, 92.14% and 92.70% accuracy in 10-fold cross-validation, jackknife and independent testing respectively. StackDPP has the best independent test results, compared to the state-of-the-art methods. As its performance does not degrade between cross-validation and independent testing, it is expected that the model has been able to capture the general essence of DNA-BPs and can successfully discriminate between DNA-BPs and non-DNA-BPs when presented with novel protein sequences.

The rest of the paper is organized as follows. Section “Materials and methods” describes the materials and methods used for our research work. Our experimental results and relevant discussions are provided in section “Results and discussions”. Finally, section “Discussion and conclusion” concludes the paper.

## Materials and methods

In this section, we describe the tools and techniques that we have used for dataset preparation, protein sample representation for machine learning pipeline, feature extraction and selection, model training, performance evaluation, etc.

**Fig. 1:**
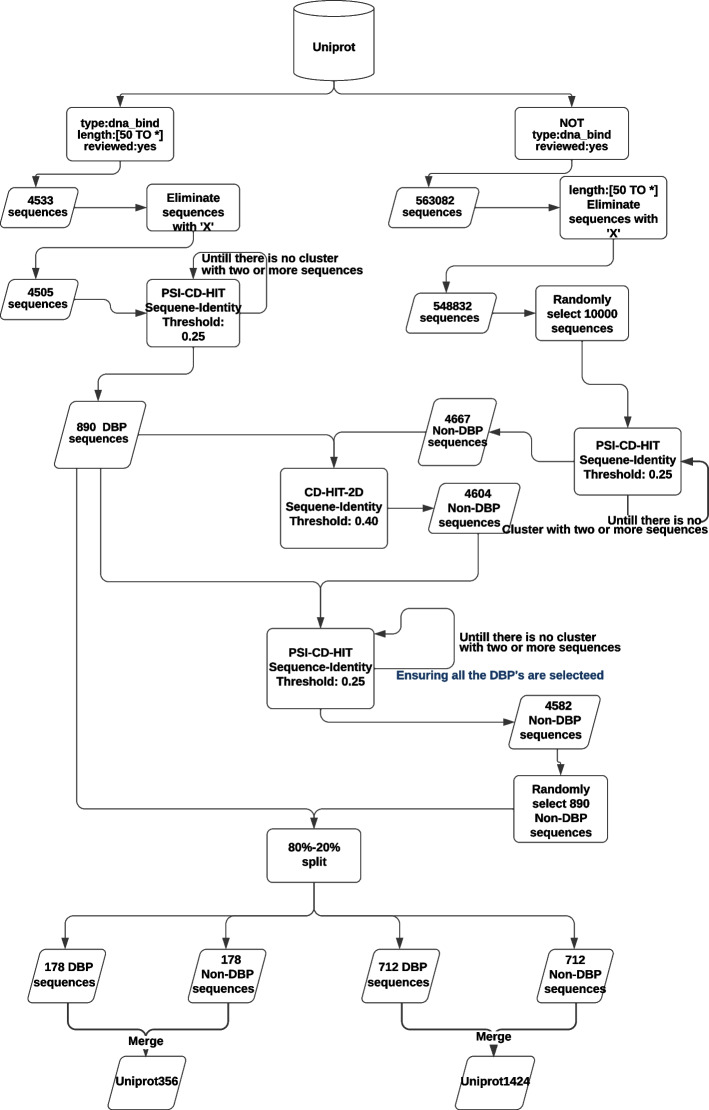
Workflow of dataset preparation

### Dataset

Building a high quality dataset is imperative for generating a robust and accurate ML-based prediction model. We investigated the widely used datasets PDB1075 and PDB186, which were respectively prepared by Chou et al. [12] and Liu et al. [13]. In our research, we identified several issues with these datasets. Therefore, we decided to create new benchmark datasets UNIPROT1424 and UNIPROT356. Detailed workflow of our dataset preparation process can be visualized in Fig. 1, which is briefly described below.

We have collected DNA-BPs and non-DNA-BPs from UniProt [22]. We have only worked with the manually reviewed proteins using UniProtKB/Swiss-Prot [23]. We then applied the following filters:

1. Discard proteins with $length \le 50$ (might be fragment)
2. Discard proteins with any residue labeled as ‘X’ (unknown residue)
3. Ensure sequence similarity threshold of $25\%$ using PSI-CD-HIT variant of CD-HIT [24] within the sets of DNA-BPs and non-DNA-BPs.
4. Ensure sequence similarity threshold of $25\%$ using PSI-CD-HIT variant of CD-HIT between the sets of DNA-BPs and non-DNA-BPs.

We have used default values for all parameters in CD-HIT, and varied the sequence identity as per our needs.. From each cluster produced by CD-HIT, we kept the longest sequence. A point to be noted here is that CD-HIT might need multiple runs to fully ensure the sequence similarity threshold. The output of $i^{th}$ run is used as the input of $(i+1)^{th}$ run. This is because CD-HIT places a sequence in either the best matching cluster or the first matching cluster depending on a parameter, but not in all of the matching clusters.

After applying filter 3 (as described above), we obtained 890 DNA-BPs. We aimed to retain all the DNA-BPs even after applying filter 4. For convenience, we applied filter 4 in two steps. We initially chose non-DNA-BPs having 40% or less sequence similarity with the 890 DNA-BPs by using CD-HIT-2D. 40% is the lowest threshold that can be set with CD-HIT-2D. Then we combined these DNA-BPs and non-DNA-BPs and ran PSI-CD-HIT with a sequence identity threshold of 25% and chose DNA-BPs out of every cluster containing more than one protein sequence. Finally, after applying filter 4 we picked up 890 non-DNA-BPs randomly. Then we did an 80-20% split on both the set of positive and negative samples. By combining 80% samples from both sets, we got our training dataset UNIPROT1424. The rest of the samples constituted the test set, which we named UNIPROT356.

### Protein representation

The simplest expression of a sequence of protein P is:

1 $$\begin{aligned} P = R_{1}R_{2}R_{3}...R_{i}...R_{L} \end{aligned}$$

Here L is the length of the protein primary sequence, and $R_{i}$ is the *i*-th residue. We would like to transform a protein sample from this sequential expression to a vector. But the transformation must somehow keep the sequence order information or any intrinsic patterns. The Pseudo Amino Acid Composition (PseAAC) [25] was developed to achieve this. According to the generic PseAAC notion [20], any protein sequence may be represented as a PseAAC vector as follows.

2 $$\begin{aligned} P = [\psi _{1}\psi _{2}\psi _{3}...\psi _{u}...\psi _{\Omega }]^{T} \end{aligned}$$

Here T is a transpose operator, and $\Omega$ is an integer whose value, as well as the components $\psi _{u}$ ($u = 1,2,\dots ,\Omega$), will depend on how the relevant information is extracted from the amino acid sequence of *P*, as explained in [26].

### Feature extraction

We have categorized all features into three different classes based on their origin – sequence-based features, PSSM features, and features based on SPIDER3 [27].

For the sequence-based features, we relied on Amino Acid Composition (AAC) [14, 17–20], Dipeptides Composition (DPC) [28, 29], Tripeptides Composition (TPC) [28], *n*-gapped-dipeptides (nGDip) [17, 30], Position specific *n*-grams (PSN) [17], Monogram Percentile Separation (MPS) [20], Bigram Percentile Separation (BPS) [20], Nearest Neighbor Bigram (NNB) [20, 30], Dubchak [14], Dipeptide Deviation from Expected Mean (DDE) [31], Grouped Amino Acid Composition (GAAC) [32], Grouped Dipeptide Composition (GDPC) [33], Grouped Tripeptide Composition (GTPC) [33], *n*-gapped Amino Acid Group Pair (nGAAGP) [33], Composition Transition Distribution (CTD) composition descriptor (CTDC) and CTD transition descriptor (CTDT) [34], Conjoint Triad (CTriad) [35], *k*-Spaced Conjoint Triad (KSCTriad) [35], Sequence-Order-Coupling Number (SOCNumber) [36, 37], Quasi-sequence-order (QSOrder), Pseudo-Amino Acid Composition (PAAC) [33], Amphiphilic Pseudo-Amino Acid Composition (APAAC) [35], K-Nearest Neighbor for peptides (KNNpeptide) [38], Moran Correlation (Moran) [39], Geary Correlation (Geary) [40] and Normalized Moreau-Broto Auto-correlation (NMBroto) [41]. Several of these features have successfully been used in the very problem of DNA-BP prediction. For example, AAC, DPC, TPC, nGDip, PSN were used in DPP-PseAAC [17]. MPS, BPS, NNB in [20], Dubchak in iDNAProt-ES [14], QSOrder in [18]. The rest have been used in various other protein related prediction problems in literature. This motivated us to experiment with these sequence-based features in StackDPP as well.

Again, for capturing the PSSM features our preference was Local Pse-PSSM (L-Pse-PSSM) [11] ($n = 1,2, 3, 4, 5$ and $\lambda = 8$ was used), PSSM Bigram (bi-PSSM) [14], PSSM 1-lead Bigram (1-bi-PSSM) [14], PSSM Composition (comp-PSSM) [14], PSSM Auto-Covariance (aCov-PSSM) [14], PSSM Segmented Distribution (segD-PSSM) [42].

Finally, for the SPIDER3 based features, we used Secondary Structure Occurrence (occ-SS), Secondary Structure Composition (comp-SS), Accessible Surface Area Composition (ASA), Torsional Angles Composition (com-TA), Structural Probabilities Composition (com-SP), Torsional Angles Bigram (bi-TA), Structural Probabilities Bigram (bi-SP), Torsional Angles Auto-Covariance (aCov-TA), Structural Probabilities Auto-Covariance (aCov-SP), Half sphere exposure (HSE).

**Fig. 2:**
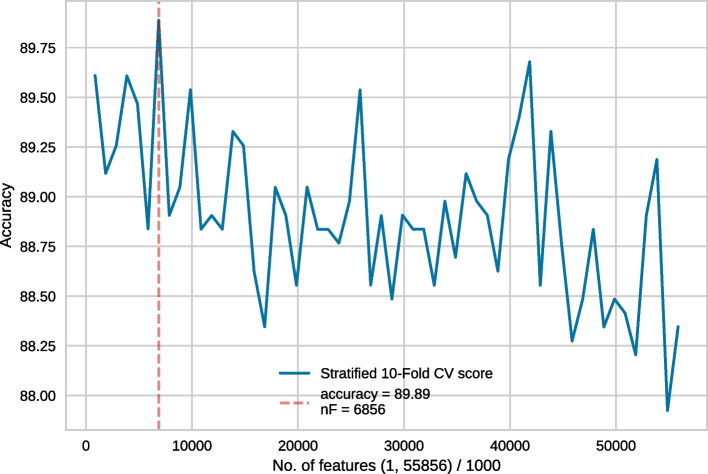
Feature selection conducted on the full set of 55856 features. In each recursive step, 1000 features were eliminated

**Fig. 3:**
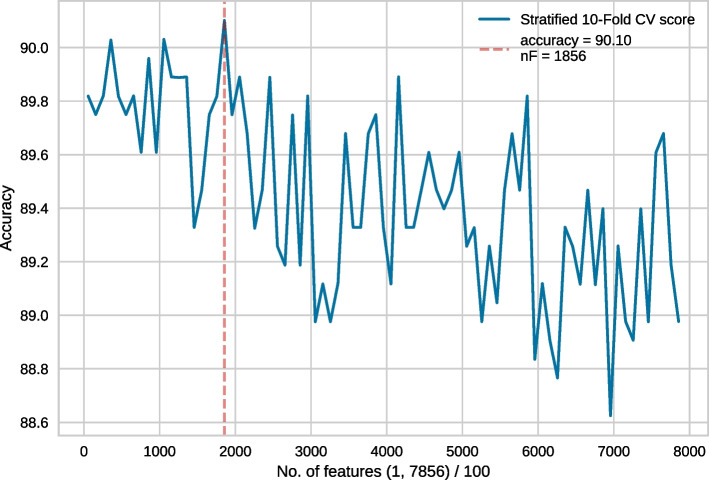
Feature selection conducted on the top 7856 features. In each recursive step, 100 features were eliminated

**Fig. 4:**
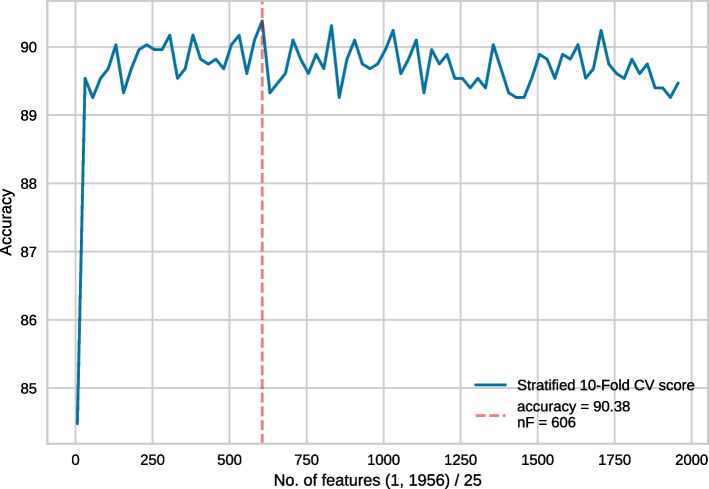
Feature selection conducted on the top 1956 features. In each recursive step, 25 features were eliminated

**Fig. 5:**
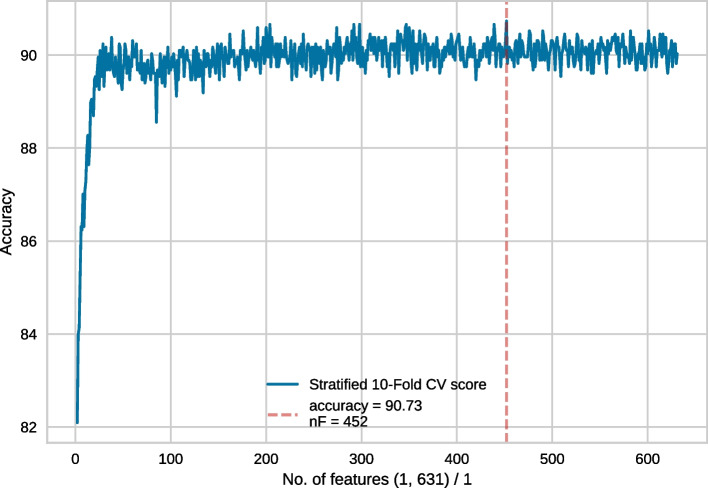
Feature selection conducted on the top 631 features. In each recursive step, 1 feature was eliminated

### Feature selection

The size of our feature vector (55856) precluded comprehensive training. We had to use feature selection to condense our feature vector into an ideal set. We used Recursive Feature Elimination with cross-validation (RFECV) for feature selection. RFECV automatically selects the best feature subset and the chance of overfitting is reduced due to internal cross-validation. We have used a Random Forest classifier for RFECV. We have used stratified *K*-Fold strategy for splitting our data into training and validation sets. In our methodology, the value of *K* is 10.

Generally, in each step of RFECV, one feature gets eliminated. However, considering our enormous feature vector, discarding a single feature at a time would take a long time. Thus, following [17] we took the following steps.

1. Feature selection was conducted on the full set of 55856 features. In each recursive step, 1000 features were eliminated. The best model performance was obtained for 6856 features. This is shown in Fig. 2.
2. We then conducted another feature selection experiment with a more granular elimination, i.e. 100 features were eliminated in each recursive step. Since 6856 features produced the best result in the earlier approach, we wanted to use slightly more features than that, hence 7856 top features were chosen for this round of RFECV. In this case, the best result was obtained for 1856 features (Fig. 3).
3. Following the same strategy, we then used the top 1956 features with an elimination step of 25 and the best performance was obtained for 606 features (Fig. 4).
4. Finally, we used the top 631 features, with 1 feature being eliminated in each recursive step. Finally, the best performance was obtained for 452 features (Fig. 5). Subsequently, we have referred to this optimal set of features as **rf452**.

**Table 1:** Result of 10-fold cross-validation on rf452 using several classifiers

Classifier	ACC (%)	SN (%)	SP (%)	MCC
Decision tree	84.27	85.53	83.00	0.6874
Logistic Regression	88.35	89.75	86.94	0.7682
Random Forest	90.03	88.76	91.30	0.8025
SVC (RBF)	91.01	90.45	91.57	0.8213
SVC (RBF, tuned)	**91.96**	91.72	**92.28**	**0.8412**
SVC (linear)	86.03	87.64	84.41	0.7224
SVC (polynomial)	89.46	**93.68**	85.25	0.7935
SVC (sigmoid)	88.83	89.61	88.06	0.7782
Extra Tree	90.81	90.31	91.29	0.8167
Gaussian Naive Bayes	88.76	89.74	87.78	0.7772
Adaboost	87.22	88.61	85.82	0.7462
Linear Discriminant Analysis	87.36	89.32	85.39	0.7484
*K*-nearest neighbour	89.39	91.01	87.78	0.7895
Bagging classifier	89.25	86.93	91.58	0.7873
Bagging with SVC (RBF)	91.08	90.73	91.43	0.8226

SVC: Support Vector Classifier. For each performance metric, the best result is shown in **bold-face**

### Choice of predictor

We have run 10-fold cross-validation on the rf452 feature set using several classifiers (Table 1). The result of the Support Vector Classifier (SVC) with Radial Basis Function (RBF) kernel was the best. Therefore we performed hyperparameter tuning on SVC with RBF kernel to further improve the performance. We achieved good 10-fold cross-validation performance with $C=5.445$ and $\gamma$=0.00237. We did the tuning by Grid Search using stratified 10-fold cross-validation. In Table 1, the result after hyperparameter tuning is shown as SVC (rbf, tuned).

**Fig. 6:**
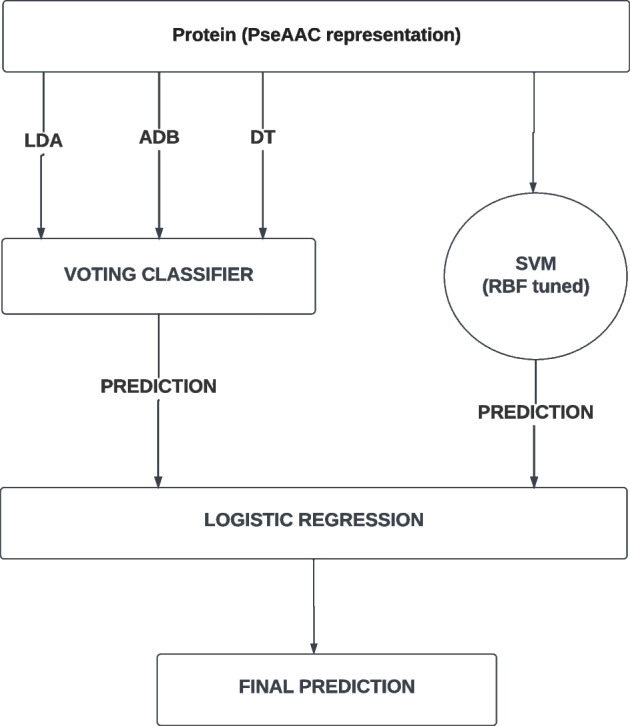
Stacking ensemble architecture of our proposed model

The cross-validation result of several classifiers was quite close to that of SVC (rbf, tuned). So we decided to ensemble some of the methods to increase the prediction performance further. We used *mlxtend library* to estimate the bias and variance of our SVC (rbf, tuned) model. Using the Mean Square Error (MSE) strategy we got an average bias and variance approximation of 0.0728 and 0.0170 respectively. As these values are reasonably low, we did not consider applying bagging or boosting. Instead, to improve the predictive quality, we decided to implement a stacking ensemble technique. We calculated the Pearson product-moment correlation of the prediction of different classifiers using the predicted probability for of positive class (Table 2). We chose three classifiers that are the least correlated – Decision Tree, AdaBoost, and Linear Discriminant Analysis (LDA). But these predictors had worse performance than SVC with the RBF kernel, as can be seen from Table 1. So we put these three classifiers within a *Voting Classifier (VC)* and added SVC (RBF, tuned) alongside it, as another base estimator. Finally, we used the logistic regression classifier as the meta-layer classifier. This architecture is shown in Fig. 6. We have named our prediction model *Stacking Ensemble Model for DNA-binding Protein Prediction*, or *StackDPP* in short.

**Table 2:** Correlation between different classifiers based on 10-fold cross-validation results

	DT	LR	RF	SVC	SVC	SVC	SVC	SVC	EXT	GNB	ADB	LDA	KNN	BG	BG
				(RBF)	(RBF, tuned)	(linear)	(poly)	(sigmoid)							(SVC-RBF)
DT	1.00	0.68	0.78	0.77	0.76	0.66	0.74	0.74	0.78	0.74	0.58	0.66	0.75	0.78	0.77
LR	0.68	1.00	0.85	0.89	0.90	0.96	0.85	0.87	0.86	0.82	0.69	0.85	0.85	0.82	0.89
RF	0.78	0.85	1.00	0.97	0.95	0.84	0.94	0.94	0.99	0.92	0.74	0.85	0.94	0.96	0.97
SVC (RBF)	0.77	0.89	0.97	1.00	0.99	0.86	0.94	0.95	0.97	0.93	0.73	0.88	0.95	0.93	1.00
SVC(RBF, tuned)	0.76	0.90	0.95	0.99	1.00	0.87	0.92	0.94	0.95	0.90	0.73	0.88	0.94	0.92	0.99
SVC (linear)	0.66	0.96	0.84	0.86	0.87	1.00	0.84	0.85	0.84	0.79	0.70	0.82	0.82	0.81	0.86
SVC (poly)	0.74	0.85	0.94	0.94	0.92	0.84	1.00	0.94	0.95	0.89	0.74	0.82	0.93	0.91	0.94
SVC (sigmoid)	0.74	0.87	0.94	0.95	0.94	0.85	0.94	1.00	0.94	0.90	0.73	0.84	0.92	0.91	0.95
EXT	0.78	0.86	0.99	0.97	0.95	0.84	0.95	0.94	1.00	0.92	0.74	0.85	0.95	0.95	0.97
GNB	0.74	0.82	0.92	0.93	0.90	0.79	0.89	0.90	0.92	1.00	0.68	0.80	0.91	0.88	0.93
ADB	0.58	0.69	0.74	0.73	0.73	0.70	0.74	0.73	0.75	0.68	1.00	0.65	0.71	0.70	0.74
LDA	0.66	0.85	0.85	0.88	0.88	0.82	0.82	0.84	0.85	0.80	0.65	1.00	0.84	0.82	0.88
KNN	0.75	0.85	0.94	0.95	0.94	0.82	0.93	0.92	0.95	0.91	0.71	0.84	1.00	0.91	0.95
BG	0.78	0.82	0.96	0.93	0.92	0.81	0.91	0.91	0.95	0.88	0.70	0.82	0.91	1.00	0.93
BG (SVC-RBF)	0.77	0.89	0.97	1.00	0.99	0.86	0.94	0.95	0.97	0.93	0.74	0.88	0.95	0.93	1.00

DT: Decision tree, LR: Logistic Regression, RF: Random Forest, SVC: Support Vector Classifier, EXT: Extra tree, GNB: Gaussian Naive Bayes, ADB: Adaboost, KNN: *K*-nearest neighbour, BG: Bagging classifier

### Predictor evaluation

We have used widely used performance metrics for evaluating our proposed predictor. These are accuracy (ACC), sensitivity (SN), specificity (SP), precision (PREC), F1-score (F1) and Matthew’s correlation coefficient (MCC). Let *TN*, *FN*, *TP* and *FP* respectively be the number of true negative, false negative, true positive, and false positive samples. Then the aforementioned metrics can be defined as follows.

3 $$\begin{aligned} ACC= & {} \frac{TP+TN}{TP+TN+FP+FN} \end{aligned}$$

4 $$\begin{aligned} SN= & {} \frac{TP}{TP+FN} \end{aligned}$$

5 $$\begin{aligned} SP= & {} \frac{TN}{TN+FP} \end{aligned}$$

6 $$\begin{aligned} PREC= & {} \frac{TP}{TP+FP} \end{aligned}$$

7 $$\begin{aligned} F1= & {} \frac{2TP}{2TP+FP+FN} \end{aligned}$$

8 $$\begin{aligned} MCC= & {} \frac{(TP\times TN)-(FP\times FN)}{\sqrt{(TP+FP)\times (TP+FN) \times (TN+FP) \times (TN+FN)}} \end{aligned}$$

Additionally, we also assessed the area under receiver operating characteristic curve (AUROC or AUC in short) and area under precision-recall curve (AUPR in short).

### Statistical test

We have used Friedman non-parametric statistical test (FMT) [43] to determine whether the results in the independent tests are statistically significant. The Friedman test is the non-parametric test for analyzing differences in multiple methods across multiple datasets. It does not assume any particular distribution of the data. All the methods are ranked in each dataset, which is then averaged to produce the average ranking. Lower rank indicates a better performer. The Friedman test was performed based on the accuracy of the predictors, with the significance level, $\alpha = 0.05$. Post hoc Holm test was conducted to perform the paired comparisons. Notably, for statistical testing, we have first bootstrapped the independent test set to produce 20 replicates and produced independent test results from StackDPP as well as several state-of-the-art methods in each replicate.

## Results and discussions

In this section, we report our analysis of the existing benchmark datasets PDB1075 and PDB186, which paved the way for creating a new benchmark dataset. We then show reproduced results of a select few state-of-the-art DNA-BP predictors. We also compare the performance of StackDPP with these state-of-the-art methods, retrained on the new benchmark dataset.

### A need for new benchmark datasets

The widely used benchmark training set PDB1075, and independent test set PDB186 were created in 2013-2014 [12, 13]. Since then a lot of protein sequences have been added to the different protein databases. It was therefore high time that a new, enhanced dataset is created for the DNA-BP prediction problem. Besides, we have found several issues in the aforementioned datasets, as described below.

Within PDB1075, only 1071 of the 1075 sequences are unique, rest are duplicate sequences despite having unique ids. On the other hand, between PDB1075 and PDB186 datasets, there are 42 proteins common by id, and 79 proteins common by sequence. Having common sequences in the training and test sets is not desirable as the test results would overestimate the quality of the predictor. As PDB1075 and PDB186 had many sequences in common, the standard training procedure with these two datasets was to train with PDB1075 for cross-validation. And for independent testing, researchers would retrain their models with reduced PDB1075, which includes only those sequences of PDB1075 that have sequence similarity less than or equal to 25% with sequences of PDB186. This process is time-consuming and there is published work (e.g. iDNAProt-ES [14]) that has missed this important step. Also, there remains a chance of error when producing reduced PDB1075 to eliminate duplicate sequences with PDB186. For example Rahman et al. [17] received 1035 sequences form Wei et al. [11] as reduced PDB1075. Though these 1035 sequences do not have any repeated sequences within themselves, there are 42 common protein sequences between this dataset and PDB186.

While preparing the PDB1075 dataset, Liu et al. [12] ensured 25% sequence similarity threshold within the positive and negative classes using PISCES [44]. However the authors did not ensure the same for the *between class* sequences. This is another limitation of this dataset.

For these reasons, we decided to create a new benchmark dataset. We have avoided repeated sequences and ensured a sequence similarity threshold of 25% among all 1424+356=1780 protein sequences in our prepared benchmark dataset.

Recently Hu et al. [21] prepared another benchmark dataset which has 17151 DNA-BPs in the training set, which is quite a large number. While the authors have mentioned that they only included manually reviewed protein sequences from UniProt, our investigation clearly shows that there is not as many reviewed DNA-BPs in that database. Therefore there is some concern about this dataset. The authors also failed to ensure less than 25% sequence similarity among the sequences. When we tried to analyze the quality of their dataset, we made the following observations.

- If we ensure 25% sequence similarity threshold in the original set of 17151 DNA-BPs and 17151 non-DNA-BPs, by using PSI-CD-HIT, we are left with only 6172 protein sequences, which is just 17.99% of the original dataset. Among these 6172 proteins, there are 3564 positive samples and 2608 negative samples.
- If we ensure 25% sequence similarity threshold in test set of 10000 DNA-BPs and 10000 non-DNA-BPs, we get only 3128 (15.64%) sequences – 2163 positive, 965 negative samples.
- Then we merged these two reduced sets and ensured a 25% sequence similarity threshold to get 6727 protein sequences of which 4082 are positive and 2645 are negative samples. So, overall after ensuring 25% sequence similarity, we have gotten only around 12.39% of the original set of sequences. This represents that the sequences were highly similar.

Based on the above analysis, it is clear that even though the size of Hu et al.’s dataset is quite large, there are questions about the quality of the data. Therefore, we chose not to use this dataset and proceeded with the preparation of a new benchmark dataset (i.e. UNIPROT1424 and UNIPROT356) as mentioned before.

**Table 3:** Cross-validation (CV) performance of our reproduction of state-of-the-art models using the PDB1075 dataset

Models	ACC(%)		SN(%)		SP(%)		MCC	
	P	R	P	R	P	R	P	R
DPP-PseAAC [17]	95.91	96.59	94.10	94.67	97.64	98.36	0.98	0.93
LocalDPP [11]	79.10	77.85	84.80	81.34	73.60	74.54	0.59	0.56
Adilina et al. [20](Group)	70.21	72.43	61	68.32	79.7	76.84	0.41	0.45
Adilina et al. [20](RFE)	71.04	71.12	62	69.44	79.9	77.81	0.43	0.44
iDNAProt-ES [14]	90.18	88.21	90.38	90.39	90	86.05	0.94	0.76

For the predictors of [20], 10-fold CV results are cited; for others we have shown the jackknife CV results. The column *P* represents results collected from the corresponding publications. The column *R* represents our reproduced results of the corresponding models

**Table 4:** Independent testing performance of our reproduction of state-of-the-art models using the PDB186 dataset. The column *P* represents results collected from the corresponding publications

Models	ACC(%)		SN(%)		SP(%)		MCC	
	P	R	P	R	P	R	P	R
DPP-PseAAC [17]	77.42	75.81	83.87	83.87	70.97	67.74	0.79	0.52
LocalDPP [11]	79.00	72.50	92.50	86.02	65.60	59.14	0.63	0.47
Adilina et al. [20](Group)	82.26	79.04	95.0	86.02	69.90	61.30	0.67	0.62
Adilina et al. [20](RFE)	76.88	77.95	77.00	93.55	76.9	62.37	0.55	0.58
iDNAProt-ES [14]	80.64	72.04	81.31	83.87	80.00	60.21	0.84	0.46

The column *R* represents our reproduced results of the corresponding model

### Reproduced results of state-of-the-art models

In this work, we have prepared a new benchmark dataset and trained a new predictor on this dataset. To compare the performance of our model with the state-of-the-art models on the right footing, it became necessary to re-train select few models on the new dataset. For some prior work, training scripts were available which could be used to retrain the model, with minor modifications. For others, we had to build the model from scratch following the description in the related publication.

DPP-PseAAC [17], iDNAProt-ES [14] and the model proposed by Adilina et al. [20] applied RFE for feature selection. But in RFE, the estimator can see the whole of the training dataset, which can produce overfitting during the ranking process. To avoid this we have instead used RFECV, which takes longer but is expected to reduce the chance of overfitting.

The source code of DPP-PseAAC [17] was available. So we used the scripts to retrain the model in the new benchmark dataset. For LocalDPP [11] we did not find the source code. So we re-implemented it using python3 using the scikit-learn library. It is to be noted that the authors worked in Weka [45]. Also, we generated PSSM using the UniRef90 database from https://www.uniprot.org/downloads, while the authors had used the nrdb90 database. For iDNAProt-ES [14], we collected the source code for feature selection from the authors. In this case, too, we have used the PSSM generated using the UniRef90 database. We have also used SPIDER3 [27] instead of SPIDER2 [16].

For the model proposed in [20], we collected the source code from the publicly shared repository. For reproducing the reported results, we have exactly followed their methodology. But when experimenting with UNIPROT1452, we increased the number of features removed in each recursive step initially. After reducing the number of features this way, we finally used 1 feature elimination in each recursive step to produce the final set of features. This was done due to resource constraints—a single run of recursive feature elimination according to their implementation was taking more than twelve days to complete.

A comparison of the published results of the different state-of-the-art models and results obtained in our attempted reproduction is given in Tables 3 and 4. The reproduced results are reasonably close to the published results. Therefore we feel comfortable that we have the training scripts for these models at our disposal which could be used to retrain the models on the new benchmark dataset.

**Table 5:** Comparison of StackDPP with state-of-the-art methods using 10-fold cross-validation on the UNIPROT1424 dataset

Model	ACC	SN	SP	MCC
DPP-PseAAC	**0.98**	**0.99**	**0.98**	**0.97**
LocalDPP	0.90	0.90	0.91	0.81
Adilina (Group)	0.86	0.84	0.88	0.72
Adilina (RFE)	0.87	0.86	0.89	0.75
iDNAProt-ES	0.94	0.94	0.94	0.88
StackDPP	0.92	0.92	0.92	0.84

The best value for each metric is shown in bold-face

**Table 6:** Comparison of StackDPP with state-of-the-art methods using jackknife cross-validation on the UNIPROT1424 dataset

Model	ACC	SN	SP	MCC
DPP-PseAAC	**0.99**	**0.99**	**0.98**	**0.97**
LocalDPP	0.90	0.90	0.90	0.80
Adilina (Group)	0.86	0.83	0.89	0.72
Adilina (RFE)	0.87	0.85	0.88	0.74
iDNAProt-ES	0.95	0.94	0.95	0.90
StackDPP	0.92	0.92	0.91	0.84

The best value for each metric is shown in bold-face

**Table 7:** Comparison of StackDPP with state-of-the-art methods using independent testing on the UNIPROT356 dataset

Model	ACC	SN	SP	MCC	PREC	F1	AUROC	AUPR
DPP-PsseAAC	0.83	0.81	0.85	0.67	0.85	0.83	0.83	0.87
Local-DPP	0.88	0.87	0.89	0.76	0.89	0.88	0.88	0.95
Adelina (Group)	0.84	0.80	0.88	0.68	0.87	0.83	0.84	0.92
Adelina (RFE)	0.85	0.85	0.86	0.71	0.86	0.85	0.85	0.92
iDNAProt-ES	0.90	**0.90**	0.89	0.80	0.89	0.90	0.90	0.96
StackDPP	**0.93**	**0.90**	**0.96**	**0.86**	**0.95**	**0.92**	**0.93**	**0.97**

The best value for each metric is shown in bold-face

### Performance comparison in the new benchmark dataset

We have shown the cross-validation performance comparison of StackDPP against state-of-the-art methods on the UNIPROT1424 in Tables 5 and 6. The former records 10-fold cross-validation results while the latter logs the results from jackknife testing. In both cases, DPP-PseAAC is the winner by a considerable margin with respect to all four performance metrics. However, as mentioned earlier, DPP-PseAAC seems to be overfitting the training dataset. This is also evident in the independent test results on the UNIPROT356 dataset.

Table 7 shows the independent test results on the UNIPROT356 dataset. The results of DPP-PseAAC degraded significantly in independent testing—there was $\approx$15% reduction in accuracy and $\approx$13% reduction in specificity from the cross-validation test results. For the rest of the predictors, the cross-validation results generalized fairly well in the independent testing. StackDPP had the best score for each performance metric.

In eukaryotic genome, around 6–7% of the genes encode proteins that are DNA-binding [1]. In a practical scenario a biologist may thus come across datasets where the expected number of DNA-BPs is very small. It is important to check whether StackDPP will perform well in the face of such heavy imbalance. Therefore, we generated a subsampled test set from UNIPROT356, comprising 12 DNA-BPs and 178 non DNA-BPs, thus keeping the amount of DNA-BPs to around 6%. We generated 20 replicates and measured the performance of StackDPP as well as the other predictors. The results, mean and standard deviation of various performance metrics, are reported in Table 8. StackDPP maintained high sensitivity and specificity. Precision of all the models degraded significantly, which is not surprising. Nevertheless, only StackDPP maintained high AUROC and AUPR.

**Table 8:** Performance of StackDPP and state-of-the-art predictors on independent test sets subsampled from UNIPROT356 to mimic real-world proportion of DNA-BPs

Model	Accuracy	Sensitivity	Specificity	MCC	Precision	F1-score	AUROC	AUPR
DPP-PseAAC	0.85±0.009	0.82±0.137	0.85±0.000	0.42±0.079	0.27±0.035	0.41±0.057	0.84±0.068	0.38±0.093
LocalDPP	0.94±0.007	0.31±0.117	0.98±0.000	0.35±0.120	0.46±0.113	0.37±0.121	0.65±0.059	0.38±0.100
Adelina (Group)	0.88±0.007	0.80±0.112	0.88±0.000	0.45±0.067	0.31±0.031	0.45±0.049	0.84±0.056	0.66±0.145
Adelina (RFE)	0.86±0.006	0.85±0.091	0.86±0.000	0.44±0.052	0.29±0.022	0.43±0.036	0.85±0.045	0.59±0.147
iDNAProt-ES	0.08±0.000	1.00±0.000	0.02±0.000	0.03±0.000	0.06±0.000	0.12±0.000	0.51±0.000	0.15±0.048
StackDPP	0.95±0.006	0.90±0.089	0.96±0.000	0.69±0.056	0.57±0.026	0.70±0.047	0.93±0.045	0.81±0.081

The results are averaged across 20 replicates. The values after ± symbol represent standard deviation

**Table 9:** Average rank of models based on the Friedman test

Model	Avg. Rank
StackDPP	1
Adilina et al. (RFE)	2.025
Adilina et al. (Group)	3.1
DPP-PseAAC	3.875
LocalDPP	5
iDNAProt-ES	6

As mentioned in the section “Materials and methods”, we have used Friedman test on the independent test accuracy of the different predictors, with the significance level, $\alpha = 0.05$. The Friedman statistic distributed according to Chi-square with $(n-1)$ degrees of freedom was 98.435714. Here *n* is the number of predictors, which is 6. *p*-value computed by the Friedman test is 4.77e$-$11. From the Chi-square distribution table, the critical value for 5 degrees of freedom is 11.07. As the Friedman test statistic value (98.435714) is greater than the critical value (11.07), the null hypothesis ($H_0$) is rejected. Table 9 summarizes the average ranking of the predictors, where StackDPP comes out on top.

**Fig. 7:**
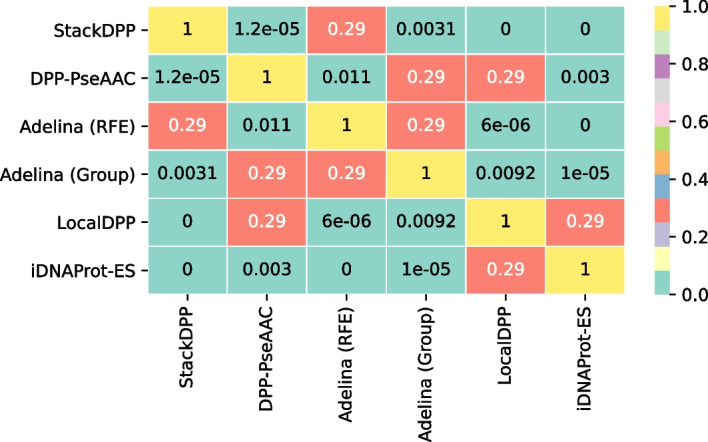
Heatmap of the adjusted *p*-values from the post hoc Holm test on the accuracy metric

Post hoc Holm test was conducted subsequently. The adjusted *p*-values for each pair of methods have been plot in a heatmap in Fig. 7. It is clear that superiority of stackDPP over rest of the methods, except for Adilina et al. (RFE) is statistically significant.

**Fig. 8:**
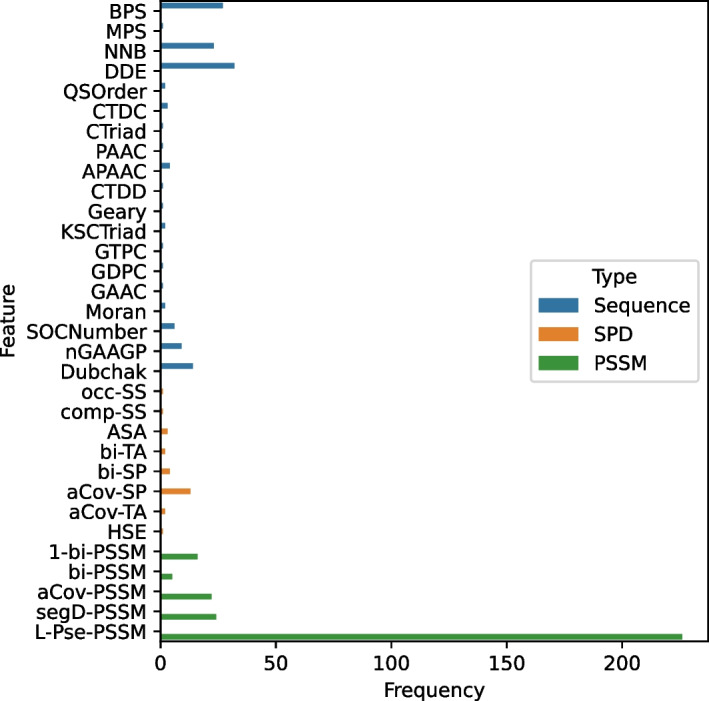
Feature Set Composition

### Feature set composition

As mentioned earlier, we explored a large number of features from three feature categories – sequence based features, PSSM based features, and SPIDER3 based features. We selected 452 top features using RFECV to train the final model. In Fig. 8 we can see the number of features of each category that we have selected in our model. Most of the selected 452 features are PSSM based. To be specific, 132 (29%) of the features are sequence based, 27 (6%) SPIDER3 based, and 293 (65%) PSSM based. This suggests that evolutionary features might be the key to successfully identifying DNA-binding proteins.

## Discussion and conclusion

In this research work, we have critically assessed the existing widely used benchmark datasets, PDB1075 and PDB186, for the DNA-binding protein prediction problem. After unraveling several problems with these datasets, we then prepared new benchmark datasets UNIPROT1424 and UNIPROT356, respectively for training and independent testing of different predictors. We have ensured that any pair of sequences from these two datasets combined have less than 25% sequence identity. We then retrained several state-of-the-art predictors on UNIPROT1424, reported their cross-validated performance, and finally tested the models using the UNIPROT356 independent test set. We have also proposed our own prediction model, named StackDPP. Our stacking ensemble based model produces at per results with state-of-the-art predictors in 10-fold and jackknife cross-validation testing. In independent testing, StackDPP outperforms all the other predictors. More importantly, its cross-validation results generalize very well in independent testing. Therefore, we strongly believe that StackDPP, which is freely and publicly available, can be successfully used in annotating novel protein sequences as DNA-binding or not. This can immensely benefit researchers in their downstream analyses.

Like DNA-binding proteins, RNA-binding proteins are also an important class of proteins to study. Many properties of known RNA-binding protein motifs are similar to those of DNA-BPs. Thus an RNA-binding protein can potentially confuse a DNA-BP predictor. Whether StackDPP is able to differentiate between the two will be something interesting to investigate in future. As there is yet another class of proteins that can bind to both DNA and RNA, dataset should be carefully curated for such an investigation. A multi-class classification problem can also be formulated to differentiate among these classes of proteins and the ones that binds neither with DNA nor RNA.

Compared to the number of sequence-known proteins, very few proteins have their structures experimentally determined. Thus researchers have tried to build predictors that do not directly rely on the strurctural informatio. However, AlphaFold 2 [46] has been very successful in computationally determining the tertiary structure of proteins with high accuracy. Therefore utilizing this information in DNA-BP classification is another important direction to explore. At the same time, recently built protein language models [47, 48] have also successfully been used in many protein attribute prediction problems. We wish to investigate these models in the context of the DNA-BPs in future as well. We also plan to build a web version of StackDPP so that researchers can schedule DNA-BP prediction jobs and get the results from a powerful server quickly.

In adddition to proposing StackDPP, an highly effective predictor of DNA-binding proteins, we have also benchmarked several existing predictors in the new dataset curated in this study. We hope that this gives the foundation for other researchers to come up with novel ideas to train their DNA-BP predictors in this dataset and benchmark their models’ performance against the state-of-the-art predictors without much hassle. Overall, StackDPP and the dataset preparation groundwork associated with it advance the frontier of research around the DNA-binding protein prediction problem considerably.

## Data Availability

The source code (python) of StackDPP and the protein sequences (FASTA file) of the proposed benchmark datasets UNIPROT1424 and UNIPROT356 are available publicly at https://github.com/HasibAhmed1624/StackDPP.
